# Diverse Functions of γδ T Cells in the Progression of Hepatitis B Virus and Hepatitis C Virus Infection

**DOI:** 10.3389/fimmu.2020.619872

**Published:** 2021-02-01

**Authors:** Wen Hou, Xiaoli Wu

**Affiliations:** ^1^ Key Laboratory for Critical Care Medicine of the Ministry of Health, Tianjin First Central Hospital, Tianjin, China; ^2^ State Key Laboratory of Medicinal Chemical Biology, Nankai University, Tianjin, China; ^3^ School of Life Sciences, Tianjin University, Tianjin, China

**Keywords:** γδ T cells, hepatitis B virus, hepatitis C virus, progression, cytokines

## Abstract

Hepatitis B virus (HBV) and hepatitis C virus (HCV) infections are primary risk factors for a wide spectrum of liver diseases that severely affect human health. The liver is an immunological organ that has an abundance of immune cells. Thus, various innate or adaptive immune cells are involved in the progression of HBV or HCV infection. Among those cells, a unique kind of immune cell, the γδ T cell, contributes to promoting or inhibiting the progression of liver diseases. To reveal the diverse roles of γδ T cells in HBV or HCV infection, the properties and functions of these cells in human and mouse models are analyzed. Here, we briefly describe the characteristics and functions of γδ T cells subsets in liver diseases. Then, we fully discuss the diverse roles of γδ T cells in the progression of HBV or HCV infection, including stages of acute infection, chronic infection, liver cirrhosis, and hepatocellular carcinoma. Finally, the functions and existing problems of γδ T cells in HBV or HCV infection are summarized. A better understanding of the function of γδ T cells during the progression of HBV and HCV infection will be helpful for the treatment of virus infection.

## Introduction

Hepatitis B virus (HBV) and hepatitis C virus (HCV) infections are major risk factors for a wide spectrum of liver diseases. Although most adults recover from HBV infection, about 5% of patients are unable to clear HBV and thus develop chronic HBV infection (1) and experience virus flares and long-term morbidity. Similarly, acute HCV infection can easily convert into chronic HCV infection (2). The persistent inflammatory environment in chronic HBV (CHB) or chronic HCV (CHC) infection patients is associated with the elevated expression of α-smooth muscle actin and collagen fibers in hepatic stellate cells (HSCs), which then develop into liver cirrhosis (2–4). Hepatocellular carcinoma (HCC) is a common cancer and is mainly caused by HBV or HCV infection. HCV patients show a higher probability of developing HCC than HBV patients (5).

The liver is known as an immune tolerance organ. Aside from hepatocytes and stellate cells, there are various hepatic residential immune cells, including Küpffer cells (hepatic macrophages), T cells, natural killer (NK) cells, and dendritic cells (6). These cells play crucial roles in the pathogenesis of HBV or HCV infection. During acute HBV or HCV infection, innate immune cells such as NK cells are activated and further induce antiviral function of adaptive immune cells (7). In chronic HBV and HCV infections, the liver is infiltrated with impaired antiviral T cells and activated inflammatory cells such as IL-17-producing CD4^+^ T cells that further exacerbate liver inflammation (8, 9). Moreover, other hepatic immune cells, including regulatory T cells and myeloid-derived suppressor cells (MDSC), prompt the pathogenesis of chronic HBV or HCV infection, liver cirrhosis, or even liver cancer (10). The proportion of hepatic γδ T cells in hepatic T cells in humans and mice is found to be 15%–25% and 4.5%, respectively (6, 11), indicating the crucial role of these cells in liver diseases. However, the current understanding of the function of γδ T cells compared with other immune cells in HBV or HCV infection is limited.

γδ T cells, as the bridge of innate and adaptive immunity, play critical roles in various diseases, including liver diseases, infections, and cancer. γδ T cells can be divided into different subsets through γ and δ TCR chains. Based on δ TCR chains, human γδ T cells can mainly be separated into Vδ1 (in peripheral blood or organs), Vδ2 (peripheral blood dominant γδ T cells, usually combined with Vγ9), and Vδ3 (in intestine and lamina propria) T cell subsets. Based on γ TCR chains, mouse γδ T cells can be divided into Vγ1, Vγ4, Vγ5, Vγ6, and Vγ7 T cell subsets (12). In liver diseases, hepatic γδ T cells usually include Vγ1, Vγ4, and Vγ6 in mice and Vδ1, Vδ2, and Vδ3 in humans (13–15). These cells can produce cytokines such as IFN-γ, TNF-α, IL-17, and IL-22, as well as express cytotoxic and regulatory molecules such as Granzyme B (GrB), perforin, NK receptor, and Toll-like receptors (16). γδ T cells play different roles in the pathogenesis of HBV and HCV infections. In acute HBV infection, human γδ T cells are activated and exhibit antiviral functions by secreting IFN-γ and TNF-α. During other stages of HBV and HCV infections (chronic infection, liver cirrhosis, and HCC), these cells can inhibit or promote progression of the diseases. Surprisingly, different subsets of γδ T cells play contradictory roles in the same stage of liver infection. For example, in chronic HBV infection, human Vδ2 T cell subsets inhibit HBV infection progression by inhibiting Th17-induced liver damage (17). However, human CD4^-^CD8^-^ γδ T cell (18) and mouse IL-17-producing Vγ4 T cell (19) subsets are found to inhibit the function of T cells and promote HBV infection in CHB patients and an HBV mouse model. Similar contradictory functions are also observed in other stages. In HCC, human Vδ2 T cells, which can be activated and proliferate *in vitro* (20), are used in the clinic to prolong the survival time of HCC patients (21).

To determine the precise role of these cells, we summarize the functions of different human and mouse γδ T cells subsets in the different stages of HBV and HCV infections. Moreover, we indicate the opportunities and challenges in clinical application of γδ T cells.

## Role of γδ T Cells in Acute and Chronic HBV Infection

During human acute HBV infection, about 5% of adult patients progress to chronic hepatitis B infection, whereas the rest go through a self-limited process that results in recovery (1). Accumulating data have demonstrated that different outcomes of HBV infection are associated with the intensity of antiviral immune responses (22). As shown in our previous study, the numbers of γδ T cells increase in liver tissue, but decrease in the peripheral blood of acute hepatitis B (AHB) patients (3). These peripheral γδ T cells are highly activated and terminally differentiated into memory phenotype, which has increased cytotoxic capacity and enhanced antiviral activity. Interestingly, in asymptomatic HBV infection patients, the frequencies of peripheral Vδ1 and Vδ2 T cells are higher, and the level of peripheral IFN-γ^+^Vδ2 T cells is also significantly elevated compared to healthy controls (23). Furthermore, in an AHB infection mouse model, the number of hepatic γδ T cells significantly increases with the upregulation of HBV markers and exhibits elevated expression of the activation marker CD69, IFN-γ production, and IFN-β mRNA abundance in liver tissues (24). The above studies indicate that the antiviral function of γδ T cells in AHB patients can inhibit the progression of AHB infection.

γδ T cells display contradictory roles in CHB infection. Several studies have shown that these cells are impaired and exhibit liver protective functions to inhibit the progression of CHB infection (17). Our study and others show that the frequency of human peripheral and hepatic Vδ2 T cells is significantly lower in severe CHB patients with impaired chemotaxis (17) or degranulation (25). Although they display an active effector-memory phenotype (17), the IFN-γ or TNF-α-induced cytotoxicity of Vδ2 T cells is impaired (26) and can be reversed by IFN-α treatment *in vitro* and *in vivo* (27). In addition, *in vitro* proliferated human Vδ2 T cells can inhibit inflammatory cytokines production in pathogenic Th17 cells (17), which contributes to significant liver damage and pathology. However, a recent study indicates that the frequency of human γδ T cells and their subsets barely change and antiviral function of Vδ2 T cells is enhanced in CHB patients (28). This opposite result maybe because of the different applied standard for patient enrollment, including age, gender, and race, which would interfere the characteristics of γδ T cells (29).

However, other studies report that γδ T cells promote the progression of chronic HBV infection. By suppressing the secretion of HBV core peptide-stimulated IFN-γ and TNF-α by CD8^+^ T cells, human CD4^-^CD8^-^ γδ T cells limit T cell responses to HBV partially through NKG2A and may impede HBeAg seroconversion during antiviral therapy of CHB patients (18). Moreover, in HBV-associated acute-on-chronic liver failure (CHB-ACLF) patients, more human peripheral γδ T cells exhibit upregulation of TNF-α or IL-17 and GrB or CD107, demonstrating the participation of γδ T cells in liver injury which in turn promote the progression of liver diseases (30). Meanwhile, in an immune tolerance chronic HBV infection mouse model, IL-17-producing Vγ4 T cells recruit MDSCs into the liver and induce CD8^+^ T cell exhaustion (19).

In conclusion, IFN-γ- or TNF-α-producing γδ T cells can inhibit AHB and CHB infection, while human CD4^-^CD8^-^ γδ T cells and mouse IL-17-producing Vγ4 T cell subsets promote the progression of chronic HBV infection. The opposite roles of these cells can be attributed to the different subsets of γδ T cells and their variable cytokine production (IFN-γ, TNF-α, or IL-17).

## Role of γδ T Cells in Chronic HCV Infection

Numerous researchers have focused on the function of γδ T cells in chronic HCV (CHC) infection. The number of hepatic γδ T cells is higher in CHC patients, and Vδ1 T cells are the predominant subset of hepatic γδ T cells (31, 32). However, the number of peripheral Vγ9Vδ2 and Vδ1 T cells decrease in CHC patients compared with healthy control and asymptomatic HCV carriers (33). Moreover, in mice, the level of hepatic γδ T cells is significantly higher in HCV transgenic mice compared with wild-type mice (34). It is assumed that peripheral γδ T cells are recruited into the liver and contribute to the pathogenesis of HCV infection.

γδ T cells play different roles in the pathogenesis of CHC infection. In some studies, γδ T cells manifest their antiviral role and inhibit the progression of CHC infection. In CHC patients, the cytotoxicity of hepatic γδ T cells is higher than that of hepatic αβ T cells. This is attributable to their elevated secretion of IFN-γ, TNF-α, and IL-8 (31) and their expression of activation marker (human leukocyte antigen-DR) and memory/effector (CD62L^-^CD45RO^+^ CD95^+^) marker (32). In particular, the frequency of human hepatic IFN-γ^+^Vδ1 T cells is positively correlated with the degree of liver necroinflammation, indicating their involvement in liver pathogenesis and liver damage (32). Furthermore, the expression of CD56 and CD16 (markers of natural killer cells) increase in peripheral Vγ9Vδ2 T cells and is further enhanced in hepatic Vγ9Vδ2 T cells of CHC patients (35). In humans, after stimulation by non-peptide antigen-isopentenyl diphosphate (IPP), activated peripheral Vγ9Vδ2 T cells are associated with a dramatic reduction in HCV RNA levels. Neutralizing experiments have further revealed the function of IFN-γ in HCV clearance (36). Moreover, in a mouse model, the number of hepatic γδ T cells increases and activated CD69^+^ γδ T cells produce more IFN-γ and TNF-α during MHV (mouse hepatitis virus) infection than controls. Interestingly, those activated hepatic γδ T cells can kill MHV-infected hepatocytes *in vitro* by secreting IFN-γ and TNF-α (37).

However, several studies have indicated that human peripheral γδ T cells exhibit impaired function in CHC patients even after antiviral treatment. Human peripheral Vγ9Vδ2 T cells are activated and differentiate into effector cells with upregulated GrB and perforin expression, but have a markedly impaired capacity to produce IFN-γ in CHC patients (38). Furthermore, IFN-α treatments result in the upregulation of cytotoxic markers such as GrB, perforin, and CD107a, but not the IFN-γ production capacity of peripheral Vγ9Vδ2 T cells in CHC patients (35, 38). The above results suggest a functional dichotomy of Vγ9Vδ2 T cells in chronic HCV infections that contribute to both liver inflammation and HCV persistence. Moreover, dysfunction of γδ T cells in CHC patients has also been observed in antiviral therapy. Direct-active antiviral agents (DAAs) are widely used in the treatment of chronic HCV infection. In clinical trials, DAAs have induced minor changes in γδ T cells both in terms of numbers and in alterations of TRG and TRD repertoires 1 year after treatment (39). Although human peripheral Vγ9Vδ2 T cells display an elevated effector phenotype in sustained virologic-response HCV patients, recent DAA treatment research demonstrates that these cells show poor cytokine response and proliferative responses to antigens (40).

In summary, human and mouse hepatic γδ T cells as well as *in vitro* stimulated human peripheral Vγ9Vδ2 T cells can inhibit HCV pathogenesis. However, impaired cytokine response of peripheral Vγ9Vδ2 T cells in CHC patients contributes to HCV infection progression, even after DAA treatment. Further studies on recovery from the cytokine response impairment of Vγ9Vδ2 T cells is very important for CHC treatment.

## Role of γδ T Cells in Liver Cirrhosis and HCC

Persistent inflammation of HBV or HCV can lead to liver fibrosis and liver cirrhosis. HSCs are critical cells in the pathogenesis of liver cirrhosis. Activation of these cells promote the progression of liver cirrhosis (41). A liver cirrhosis mouse model shows different relationships between HSCs and hepatic γδ T cells. IL-17-producing CCR6^+^ γδ T cells induce apoptosis of HSCs in a FasL-dependent manner to inhibit the progression of liver cirrhosis (42). Moreover, IFN-γ-producing γδ T cells can directly kill activated HSCs and increase NK cell-mediated cytotoxicity against activated HSCs partially through a 4-1BB dependent manner (43). However, hepatocyte-secreted exosomes can activate HSCs *via* Toll-like receptor 3. These HSCs further enhance the activity of IL-17-producing γδ T cells, which exacerbates liver fibrosis and promotes the progression of liver cirrhosis (4). In view of the contradictory roles of IL-17-producing γδ T cells in the same mouse model, further studies involving patients and a virus-induced liver cirrhosis mouse model should be performed to elucidate the exact role of γδ T cells.

A recent study has shown that the increased peritumor ratio in human γδ T cells contributes to the progression and recurrence of HCC, indicating the important role of γδ T cells in HCC (44). Interestingly, γδ T cells play different roles in the pathogenesis of HCC. In several studies, γδ T cells display cytotoxicity and inhibit proliferation of tumor cells *in vivo* and *in vitro.* In HCC patients, the number of human peritumoral γδ T cells is positively related to better prognosis of HCC curative resection (45). A recent biostatistics study has shown that the increase of human tumor-infiltrated γδ T cells, which is driven by the accumulation of chemokines such as CCL4 and CCL5, is significantly positively correlated with the survival rate and negatively correlated with HCC recurrence. γδ T cells play protective roles by regulating the infiltration and differentiation of CD8^+^ T cells in HCC procession (46). Furthermore, human γδ T cells can induce the death of HCC cell lines and reverse the immune escape of HCC *in vitro* (47). Moreover, the anti-HCC function of peripheral γδ T cells, especially Vγ9Vδ2 T cells, can be further enhanced by activating agents, including histone deacetylase inhibitors (48), pyrophosphate (49), zoledronate (20), CD226 (50), and even the Chinese herb artesunate (51).

However, other studies reveal that impaired human γδ T cells or mouse γδ T cells can also contribute to the progression of HCC. In an immunosuppressed tumor microenvironment, γδ T cells show impaired IFN-γ production and degranulation (perforin and CD107a) capacity, which is attributed to the secretion of TGF-β and IL-10 by tumor-infiltrating Tregs (52). In addition, a decrease in the number and cytotoxicity of peripheral Vδ2 T cells is observed in HCC patients and possibly associated with the lack of IL-2 and IL-21 (53). The total number of γδ T cells and effector γδ T cells is significantly lower in tumors than in peritumoral tissues and non-tumor livers (52, 54). In addition, in an HCC mouse model, IL-17-producing Vγ4 T cells recruit MDSCs in a CXCL5/CXCR2-dependent manner and further suppress the anti-tumor function of CD8^+^ T cells (55).

Human peripheral Vδ2 T cells can proliferate *in vitro* and kill HCC and thus have been used in clinical immunotherapy of HCC patients. Zoledronate induces the proliferation of γδ T cells in HCC patients who exhibit upregulated expression of IFN-γ, TNF-α, GrB, perforin, and lysosome-associated membrane protein 1 (47). A clinical trial has shown that the combined use of γδ T cells, NK cells, and cytokine-induced killer (CIK) therapy significantly inhibits virus replication and prolongs the survival rate of HCV-positive HCC patients (21).

In conclusion, γδ T cells and their subsets play opposite roles in liver cancer, and their underlying mechanisms require further investigation.

## Conclusions and Perspectives

**Figure 1:**
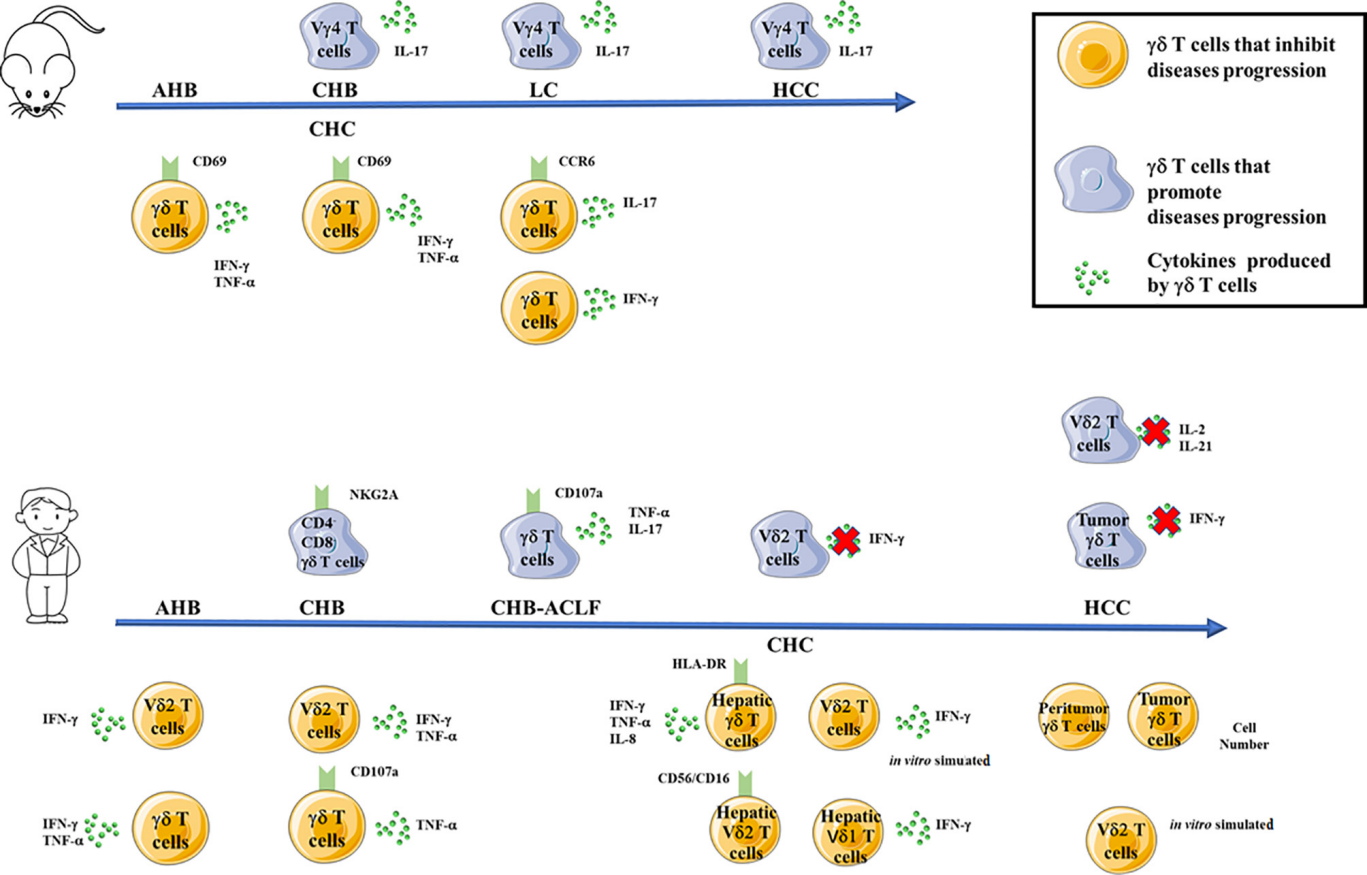
Functions of mouse (top) or human (bottom) γδ T cells and their subsets in inhibiting (yellow cells) or promoting (blue cells) the pathogenesis of HBV or HCV infection. The arrows indicate the progression of HBV or HCV infections, the red fox indicate impaired secretion of cytokines by γδ T cells and their subsets. AHB, acute hepatitis B infection; CHB, chronic hepatitis B infection; CHB-ACLF, HBV-associated acute-on-chronic liver failure; CHC, chronic hepatitis C infection; LC, liver cirrhosis; HCC, hepatocellular carcinoma.

Different subsets of γδ T cells play various roles in pathogenesis of HBV or HCV infection. Most of the mouse and human studies are summarized in **Figure 1**.

In mouse model, IL-17-producing Vγ4 T cells subsets promote the progression of CHB, LC and HCC. However, in other studies, IFN-γ and TNF-α-producing CD69^+^ mouse γδ T cells can inhibit the progression of AHB and CHC. Furthermore, IL-17-producing CCR6^+^ mouse γδ T cells or IFN-γ producing mouse γδ T cells inhibit the progression of LC. (**Figure 1**, top).

In human studies (**Figure 1**, bottom), CD4^-^ CD8^-^ γδ T cells subsets and IL-17/TNF-a^+^ γδ T cells promote the progression of CHB and CHB-ACLF patients, respectively. Impairment secretion of IFN-γ by peripheral Vδ2 T cells contributes to the progression of CHC. Moreover, impairment secretions of IL-2 and IL-21 by peripheral Vδ2 T cells and IFN-γ by tumor-infiltrating γδ T cells contribute to the progression of HCC. Contradictorily, in AHB patients, IFN-γ-producing peripheral Vδ2 T cells and IFN-γ and TNF-α-producing peripheral γδ T cells can inhibit AHB infection. In addition, IFN-γ and TNF-α-producing peripheral Vδ2 T cells and TNF-α-producing CD107a^+^ peripheral γδ T cells inhibit the progression of CHB infection. Furthermore, hepatic γδ T cells as well as *in vitro* activated peripheral Vδ2 T cells inhibit the progression of CHC infection. Furthermore, increased number of peritumor and tumor γδ T cells as well as *in vitro* activated peripheral Vδ2 T cells inhibit the progression of HCC (**Figure 1**, bottom).

Although functions of γδ T cells are summarized above, some of their roles in virus infection remain obscure. For instance, IL-17-producing Vγ4 T cells display diverse roles to influence the development of liver cirrhosis in the same mouse model. Furthermore, the role of human peripheral γδ T cells but not hepatic γδ T cells has been extensively studied. Thus, the impact of cytokine production and the functions of hepatic γδ T cell subsets in the pathogenesis of HBV and HCV infections require further investigation. The frequency and function of γδ T cells can be distinguished based on human race, age, and gender, thus these factors have to be considered in related researches (28, 29). Asian Americans display two- to three-fold higher number of peripheral Vδ2 T cells compared to non-Asian Americans (28), which in turn may partially contribute to the immune responses and outcome of virus infection. Moreover, the fate of transferred γδ T cells in the human body as well as the indication and race of liver cancer patients should be assessed to achieve better therapeutic outcomes during treatment. Last but not least, in view of their antiviral function, IFN-γ-producing γδ T cell-based therapies should be developed for patients in stages of virus infection other than HCC. Understanding the roles of γδ T cells in relation to the pathogenesis of HBV and HCV infections may facilitate in the development of γδ T cell-based therapy or γδ T cell-based targets for the treatment of virus infections.

## Author Contributions

WH wrote the main part of the review. XW wrote the Introduction and revised the manuscript. All authors contributed to the article and approved the submitted version.

## Funding

This work was supported by the Natural Science Foundation of Tianjin City (18JCZDJC34500), the State Key Laboratory of Medicinal Chemical Biology, Nankai University (2019003), and Tianjin First Central Hospital Spring Funding (CM201813).

## Conflict of Interest

The authors declare that the research was conducted in the absence of any commercial or financial relationships that could be construed as a potential conflict of interest.
